# Varieties of trust in preschoolers’ learning and practical decisions

**DOI:** 10.1371/journal.pone.0202506

**Published:** 2018-08-20

**Authors:** Annelise Pesch, Melissa A. Koenig

**Affiliations:** Institute of Child Development, University of Minnesota, Minneapolis, MN, United States of America; Swinburne University of Technology, AUSTRALIA

## Abstract

Keeping commitments to others can be difficult, and we know that people sometimes fail to keep them. How does a speaker’s ability to keep commitments affect children’s practical decisions to trust and their epistemic decisions to learn? An amassing body of research documents children’s trust in testimonial learning decisions, which can be moved in the face of epistemic and moral evidence about an agent. However, other bases for trust go largely unexplored in this literature, such as interpersonal reasons to trust. Here, we investigated how direct bids for interpersonal trust in the form of making commitments, or obligations to the listener, influence a range of decisions toward that agent. We found that 3- and 4-year-olds’ (*N* = 75) practical decisions to wait and to share were moved as a function of a person’s commitment-keeping ability, but epistemic decisions to learn were not. Keeping one’s commitments may provide children with interpersonal reasons to trust, reasons that may function in ways distinct from the considerations that bear on accepting a claim.

## Introduction

Testimony provides children with a rich source of information about the world. Much work has examined how preschool-aged children evaluate testimony, showing that children prefer to learn from agents who are more accurate [1], knowledgeable[2], and well-informed [3]. Preschoolers also monitor probabilistic or relative evidence about accuracy [4, 5], evidence about perceptual access to relevant information [6, 7], and overt evidence about expertise [8–10]. In addition, preschoolers base learning decisions on moral information [11, 12], attractiveness [13], group membership [14, 15], and age [16, 17], suggesting that children consider not only epistemic competencies when learning from others, but a variety of moral and interpersonal characteristics as well.

What does such selectivity reflect? On one view, children’s selective trust in testimony is conceptualized as a rational inference based on a range of evidence about an agent’s knowledge and intent [18–20]. This ‘evidential’ approach has launched a set of important questions regarding children’s abilities to use evidence (both positive and negative) to mitigate testimonial risks (for reviews, see[21, 22]). However, a dimension lost by focusing on the evidentiary value of testimony are the various interpersonal reasons that testimony provides to addressees to trust an agent. Thus, another way we might understand the trust elicited in selective social learning is to consider that a hearer’s decision to trust a speaker can be based in interpersonal considerations [23]. Indeed, there may be interpersonal epistemic reasons to believe someone that are not solely based in a listener’s ability to predict a speaker’s knowledge or goodwill [24]. This leaves open another sense in which children can *trust* others, taking them at their word, without knowing very much about them or even having evidence against them [25–27].

This sense of trust can be seen in our responses to obligations or commitments that others make, where trust in testimony might similarly reflect sensitivity to the interpersonal obligations or commitments that speakers present in different speech acts. A set of recent studies have found that trust is implicated in commitments to retrieve a larger second reward for a child in a delay of gratification task. Kidd, Palmeri, and Aslin [28] presented preschoolers with an experimenter who either fulfilled or broke their promise to retrieve art supplies on two occasions. Later, when tested in a traditional delay of gratification task (e.g. [29]), children in the unreliable condition were significantly less likely to wait for a second reward from that agent [28]. Thus, when confronted with an agent who stated their intent to retrieve a desired item, children’s later extensions of trust were found to be contingent on their prior interaction with that agent. A related study found that preschoolers were also less likely to wait for a larger reward in a delay of gratification task when the experimenter lied about a transgression rather than tell the truth [30]. These findings suggest that stated commitments to produce a desired item prompts children’s decisions to trust. This is further supported by recent work showing that children’s general trust views (i.e. viewing others as trustworthy) predict performance on a delay of gratification task, despite prior interaction with the agent administering the task [31]. Unclear from this work is whether this type of trust–an invitation to trust an agent to uphold her commitment to retrieve a reward–is the same type of trust that is involved when children are asked to learn new information from an agent. To our knowledge, only one study has sought to disentangle intention from outcome in an epistemic learning task. Liu, Vanderbilt, and Heyman [32] showed children an agent who either intended to help or deceive a third-party find stickers with either positive or negative outcomes. They found that 5- and 6-year-olds reasoned more about the outcome of prior testimony in their learning decisions, but took into account intention when assessing trait-relevant behavior. While this particular experiment does not answer whether the trust invited by the agent to learn was similar to the interpersonal trust invited in the delay of gratification task, it does suggest that children may be differentiating the information they use in their assessments of others.

The idea that children might base different decisions on distinct considerations is further supported by a recent body of work suggesting a set of dissociated judgments in children’s learning and practical decisions. For example, Hetherington, Hendrickson, and Koenig [33] presented preschoolers with either an antisocial in-group or prosocial out-group member. Importantly, the antisocial behavior of the in-group member reduced children’s liking of her and willingness to share with her compared to a neutral out-group member; however, it did not move decisions to seek new information from her. Likewise, when 18-month-old infants encountered an agent who incorrectly labeled a set of objects, their willingness to learn novel information was reduced but their willingness to share with that person was not [34]. When introduced to characters who differed in niceness and expertise, children based epistemic inferences on evidence of both expertise and niceness but based social inferences only on niceness [35]. Such findings suggest that children’s interpersonal reasons to trust the agent may dissociate from their epistemic reasons to believe their claims.

Do children extend different variants or forms of trust depending on the decision they are asked to make? In order to distinguish between epistemic and interpersonal extensions of trust, we presented children with learning decisions, as well as two types of practical decisions. As discussed above, delaying gratification is one type of practical decision that can be influenced by a speaker’s promise-keeping behavior. Another practical decision children make is how much and with whom to share [36, 37]. Research in behavioral economics has found that adults typically allocate slightly more than half of their money to strangers when playing a standard “trust game” [38, 39]. Children, too, share with others without any prior evidence of that person’s inclination to reciprocate [40, 41], and when asked to share a limited resource, share more with friends and familiar agents over strangers [42], suggesting that such decisions are not based in evidence about the recipient, and often moved by interpersonal considerations.

In the current study, we used commitments to elicit interpersonal trust in our target agent. Children interacted with an agent in an art project task, similar in design to Kidd et al.’s [28]. To produce violations of interpersonal trust, we presented two types of ‘bad interpersonal agents’ (one who failed to have good intentions, and one who failed to deliver good outcomes) in contrast to a good baseline agent (with good intentions, and who delivered good outcomes). We thus explored the extent to which interpersonal violations of trust–both in terms of intention and outcome–would influence children’s practical decisions (to delay gratification, to share a limited resource) and their epistemic learning decisions (to ask her for information and to endorse her claims). If interpersonal violations of trust are simply treated as inductive evidence about the reliability or goodwill of the agent, then we expect such information to broadly move children’s learning and practical decisions (consistent with [11, 12, 43, 44]). But, if interpersonal violations of trust constitute a violation of children’s interpersonal decisions to trust a speaker, then such violations might influence children’s interpersonal decisions—decisions to wait for the agent and to share with her—rather than their epistemic decisions to learn.

## Method

### Ethics statement

The Institutional Review Board at the University of Minnesota approved this research (#1603P85562). Written consent was obtained from the parents or legal guardians of the participants. Each participant verbally agreed to take part in the study.

### Participants

Seventy-five 3- and 4-year-olds participated in the study (38 Female, *M*_age_ *=* 4.05; range = 3.07–4.96). This sample size was based on having 80% power to detect a moderate effect size of *f* = .36. Children were randomly selected from a database of children who are primarily Caucasian, native English speakers from middle to high SES homes. Children were assigned to one of three between-subject conditions: *Baseline* (positive intention-positive outcome) (*n* = 25, 12 females, *M*_age_ = 4.11; range = 3.13–4.97), *Failed Commitments—Negative Outcomes* (*n* = 25, 11 females, *M*_age_ = 4.16; range = 3.08–4.88), or *Failed Commitments—Positive Outcomes* (*n* = 25, 12 females, *M*_age_ = 4.01; range = 3.24–4.88). Eight additional children were excluded due to experimenter error (*n* = 4) or failure to complete the experiment (*n* = 4).

### Procedure

**Fig 1:**
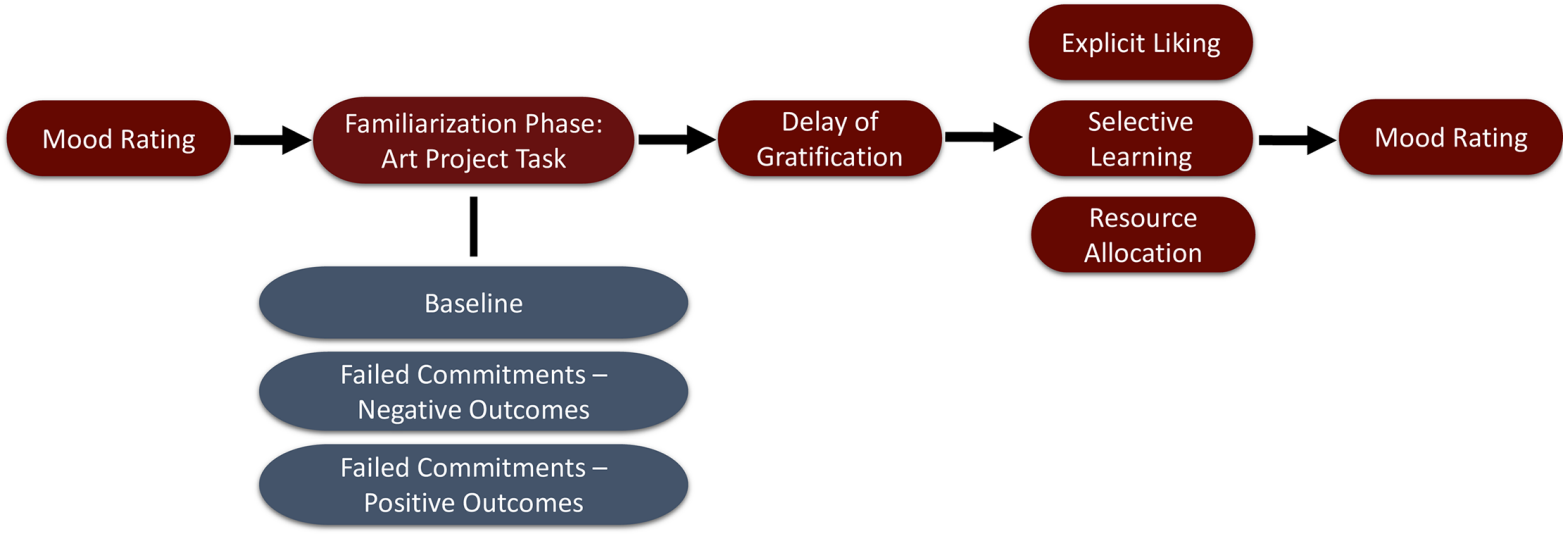
Flow chart of the procedure.

Across all conditions, Experimenter 1 (E1) first asked participants for a mood rating. Participants were then invited to the testing room to participate in an art project task. Participants’ interaction with E1 during the art project differed depending on condition. E1 then administered the delay of gratification task. Following the delay of gratification task, E1 left and Experimenter 2 (E2) entered the testing room and administered the explicit liking, resource allocation, and selective learning tasks. These tasks were a series of video clips involving two agents: E1 and an unfamiliar, neutral agent (E3). Across participants, we counterbalanced which agent was presented first, which agent spoke first, and which side of the screen the agent appeared on. After completing these tasks, the second mood rating was administered. (See Table 1 for an overview of our procedure supplemented also by Fig 1).

**Table 1:** Procedural overview.

**Art Project Task**		
**Condition**	**Intention/Outcome Statement**	
Baseline	*I really want you to have the best art supplies/sticker (leaves room)*. *Here they are*!	
Failed Commitments—Negative Outcomes	*I really want you to have the best art supplies/sticker (leaves room)*. *I’m sorry*, *but we don’t have any after all*.	
Failed Commitments—Positive Outcomes	*I really don’t want you to have the best set of art supplies/stickers because I want the best for myself (leaves room)*. *Actually*, *here’s another set of art supplies/stickers you can use*, *I saved the very best for myself but you can use these*.	
**Dependent Measures**		
**Task**	**Order of Presentation**	**Question**
Mood Rating	2 Trials: before Art Project and after EL 2	*“Circle the smiley face that matches how happy you are right now”*
Delay of Gratification	After Art Project	*“You can have this one marshmallow right now*, *or if you can wait for me to get more*, *you can have two to eat instead*.*”*
Explicit Liking (EL)	2 Trials: after Delay and after RA 3	*“How much do you like her [familiar/neutral]*?*”*
Selective Learning (ST)	6 Trials: 2 after EL 1, 2 after RA 1, and 2 after RA 2	Ask: *“Who would you like to ask*?*”* Endorse: *“Where do you think the [object] is hidden*?*”*
Resource Allocation (RA)	3 Trials: before ST 1, after ST 4, and after ST 6	*“How many coins should [neutral/familiar] get and how many coins should [neutral/familiar] get*?*”*

*Note*. E1 administered Art Project, Mood Rating 1, and Delay of Gratification. E2 administered EL, ST, RA, and Mood Rating 2. The EL, ST, and RA tasks were a series of images and video clips involving E1 and E3.

#### Mood rating (adapted from [30])

In line with the procedures used in Kidd et al. [28] and Michaelson and Munakata [30], the pre- and post-test mood ratings served to investigate whether or not the experimental manipulation affected participants’ mood. If so, this would potentially explain performance on our dependent measures. Participants were shown a six-point smiley face scale and asked to circle the face that matched how happy they were.

#### Art project task (adapted from [28])

Participants were given two opportunities to wait for a larger reward from E1. For both choices, all participants were told that they could choose to use the current supplies on the table or wait for better supplies from the other room. Following Kidd, Palmeri, and Aslin [28], participants were presented with a small set of well-used crayons (e.g. dull, wrapper peeled and dirty) in a tightly sealed jar for Choice 1 and a small star sticker inside a plastic envelope for Choice 2. The larger reward was a large art set containing new crayons, colored pencils, and markers for Choice 1 and a sleeve of attractive stickers for Choice 2.

Intention information: In the *Baseline* and *Failed Commitments—Negative Outcomes* conditions, E1 stated her positive intentions across choices 1 and 2, “I really want you to have the best set of art supplies/stickers,” before she left the room. In the *Failed Commitments—Positive Outcomes* condition, E1 stated her negative intentions across choices 1 and 2, “Actually, I really don’t want you to have the best set of art supplies/stickers because I saved the best set for myself,” before she left the room. The experimenter left the participant in the room alone for 2.5 minutes. All participants chose to wait. (See S2 File. Experimental Script: Art Project and Delay of Gratification in Supporting Information for full scripted dialog).

Outcome information: In the *Baseline* condition, the experimenter returned with the promised supplies and stated, “Here is the best set of art supplies/stickers!” In the *Failed Commitments—Negative Outcomes* condition, the experimenter returned without the promised supplies and stated, “I’m sorry, but we don’t have any other art supplies/stickers after all, why don’t you use these [points to crayons/sticker on table] instead?” In the *Failed Commitments—Positive Outcomes* condition, the experimenter returned with supplies and stated, “Actually, here is another set of art supplies/stickers. I saved the very best for myself but you can use these.” All participants were encouraged to use the supplies for approximately 3 minutes.

#### Delay of gratification task (adapted from [28])

Immediately following the art project task, E1 produced a marshmallow on a plate and gave the child the choice to eat one marshmallow right away, or wait for her to return with a second marshmallow. E1 then gathered up the materials from the art project task and left the participant alone in the room. The experimenter covertly observed the participant through a one-way window and waited until the participant had either eaten the marshmallow, became visibly upset, or 15 minutes had elapsed. In all scenarios, the experimenter returned to the room and delivered a second marshmallow to the participant, which ensured that participants received the same intention and outcome information about the experimenter within conditions before engaging in the explicit liking, resource allocation, and selective learning trials.

#### Explicit liking

Two explicit liking trials were given: once before the selective learning and resource allocation trials, and the second after (Refer to Table 1). Participants were separately shown an image of E1 and E3 and asked to rate how much they liked that agent on a six-point smiley face scale. Across participants, we counterbalanced which agent’s image they were shown first.

#### Selective learning

Participants were shown an image of an object (e.g. key) and told, *“The first thing that’s hiding is this [object]*. *I wonder where it is*!*”* Participants then saw a still image of the two agents with a different colored box in front of each one and were asked to indicate which agent they would like to ask to find out where the object was (ask trials). The agents then made conflicting claims about the location of the object (e.g. *“I think it’s in the red box”* vs. *“I think it’s in the blue box”)* and participants were asked to indicate where they thought the object was hidden (endorse trials). Participants completed six ask and six endorse trials. Each trial included a different object and a different set of colored boxes.

#### Resource allocation

Participants completed three resource allocation trials to assess their willingness to share resources with the agents. Participants were presented with two cups, each with an image of one of the agents and five paper coins. The experimenter explained to participants, *“Here are five coins for you to share*. *How many coins should this person [point to one agent] get and how many coins should this person [point to second agent] get*?*”* Participants were encouraged to place all five coins into the cups. These trials were dispersed throughout the selective learning trials: the first appearing after selective learning trial two, the second after trial four, and the third after trial six.

## Results

Preliminary analyses found no effect of gender or order, so these were collapsed within conditions for the following analyses to allow for greater statistical power.

### Mood rating

We ran a three way mixed analysis of variance (ANOVA) with condition (*Baseline* vs. *Failed Commitments—Negative Outcomes* vs. *Failed Commitments—Positive Outcomes*) and age (3 vs. 4) as the between-subjects factors, trial (trial 1 vs. trial 2) as the within-subjects factor, and mood rating as the dependent variable. There were no main effects of condition (*F*(2, 67) = 0.795, *ns*) or age (*F*(1, 67) = 2.572, *ns)*. There was a significant effect of trial (*F* (1, 67) = 5.058, *p* = .02): children’s initial mood ratings (*M* = 5.26) were higher than their second mood rating (*M* = 4.82). The absence of condition differences suggest that our measures of interest are unlikely to have been influenced by changes in mood.

### Explicit liking

Preliminary analyses found no difference between trial 1 and trial 2 for explicit liking, so these were collapsed within agent. We ran a three way mixed ANOVA with condition (*Baseline* vs. *Failed Commitments—Negative Outcomes* vs. *Failed Commitments—Positive Outcomes*) and age (3 vs. 4) as the between-subjects factors, agent (E1 vs. E3) as the within-subjects factor, and liking score as the dependent variable. There was no effect of condition (*F*(2, 68) = 1.44, *ns*) or age (*F*(1,68) = 0.16, *ns*). There was a significant effect of agent (*F*(1,68) = 14.32, *p* < .001): Children rated E1 (*M =* 4.93) as more likeable than E3 (*M* = 4.12).

### Practical decisions (delay of gratification and resource allocation)

#### Delay of gratification

**Fig 2:**
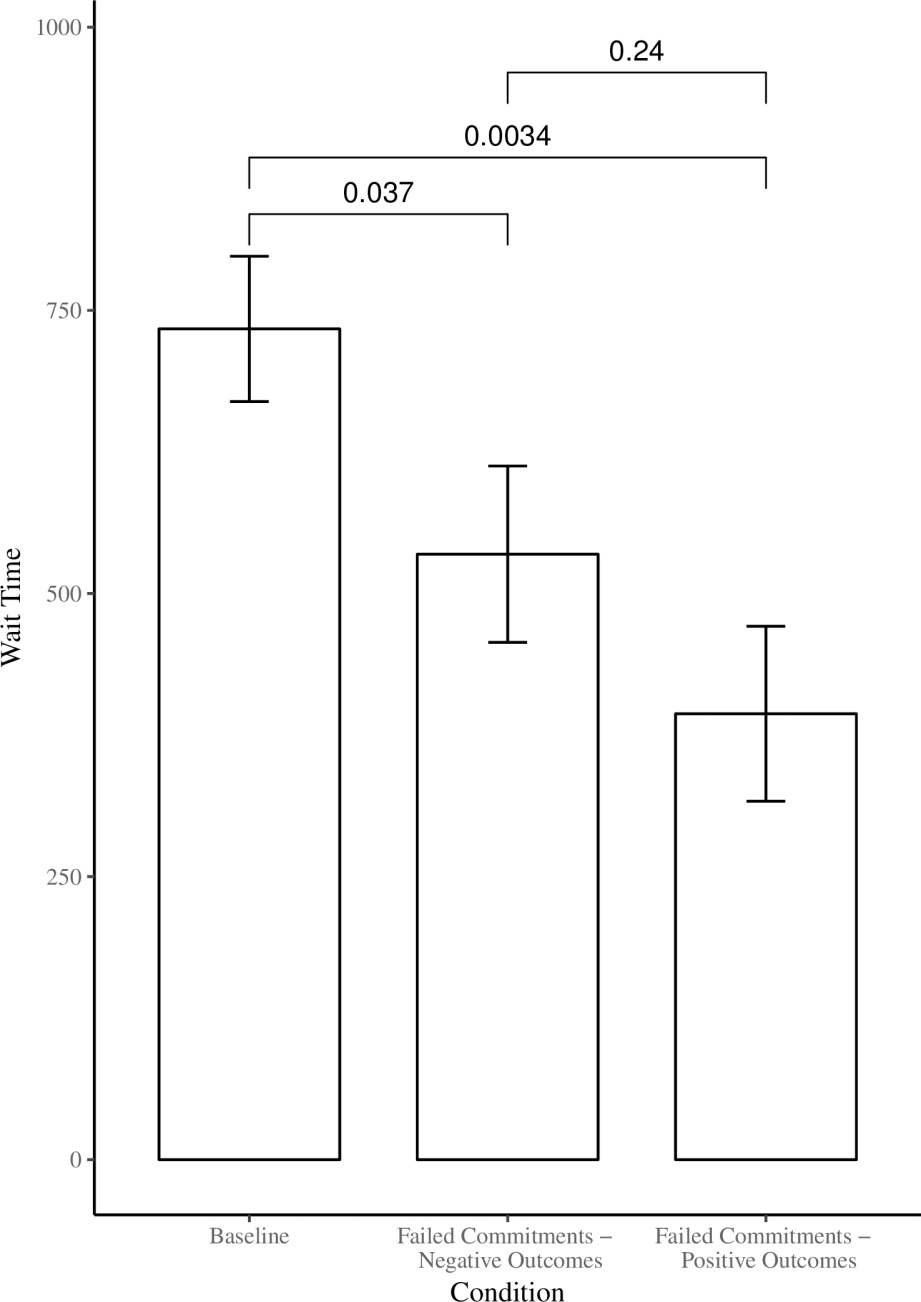
Mean wait time (max wait time = 900s) that participants waited for a second marshmallow as a function of condition. Error bars represent standard errors of the mean.

In the *Baseline* condition, children waited without eating the marshmallow for a mean duration of 733.64s whereas those in the *Failed Commitments—Negative Outcomes* condition waited 534.68s and those in the *Failed Commitments—Positive Outcomes* condition waited 393.80s (See Fig 2). Since these data were not normally distributed, a Kruskal-Wallis rank sum test was performed to test the null hypothesis that the conditions had the same distribution of wait times. The Kruskal-Wallis test showed that there was a significant difference in wait time between the three conditions, *X*^2^(2) = 9.31, *p* = 0.009. Post-hoc comparisons using Wilcoxon rank sum test revealed that children in the *Failed Commitments–Negative Outcomes* and *Failed Commitments–Positive Outcomes* conditions waited less compared to those in the *Baseline* condition (*p* = 0.03 and *p* = 0.003, respectively).

A linear regression analysis was run to test for the effect of age, controlling for condition. Age was significant, (B = 186.94, *t =* 2.26, *p* = .02), indicating that 4-year-olds (*M =* 643.8s) waited longer than 3-year-olds (*M =* 456.8s). In addition, we examined whether differences existed in the proportion of children waiting the entire 15 min without tasting the marshmallow. A chi-square test confirmed that the proportion of children waiting the full 15 minutes was significantly different between conditions (*X*^2^(2) = 10.39, *p* = 0.005). In the *Baseline* condition, 72% of children (18 out of 25) waited the full 15 minutes. The proportion of children waiting was significantly less compared to those in the *Baseline* condition in the *Failed Commitments—Negative Outcomes* condition (40% or 10 out of 25, *p* = .04) as well as in the *Failed Commitments—Positive Outcomes* condition (28% or 7 out of 25, *p* = .004).

#### Resource allocation

**Fig 3:**
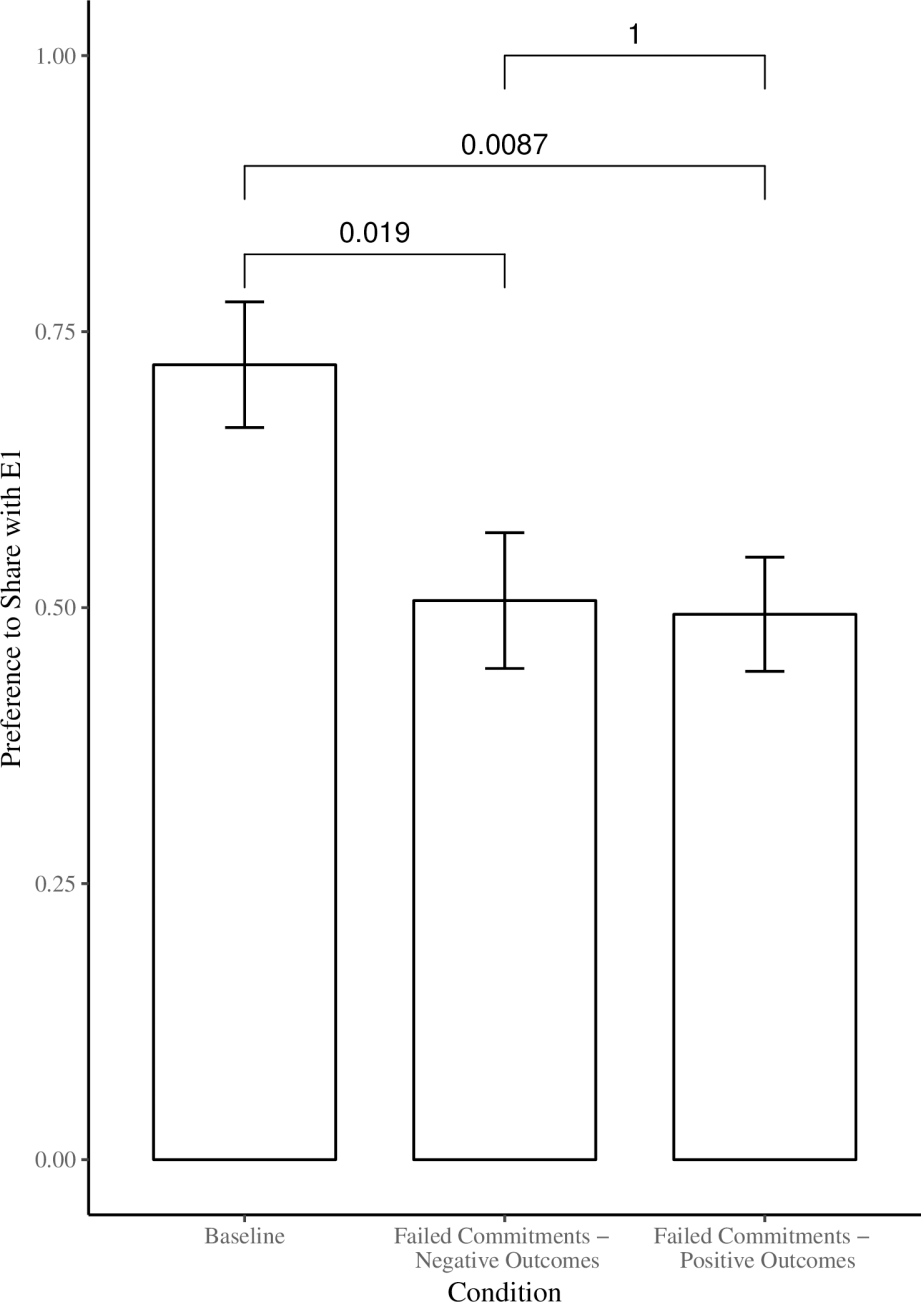
Mean proportion of times participants allocated resources to E1 (out of 3 trials) as a function of condition. Error bars represent standard errors of the mean.

A sharing score was calculated by summing the amount of trials (max = 3) on which children shared more coins (i.e. 3 or more) with E1. This score was converted to a percentage for ease of interpretation. An omnibus ANOVA was run with age (3 vs. 4) and condition (*Baseline* vs. *Failed Commitments—Negative Outcomes* vs. *Failed Commitments—Positive Outcomes*) as between-subjects factors, and sharing score as the dependent measure. We found no effect of age (*F*(1,69) = 1.87, *p* = .17). We found a main effect of condition (*F*(2,69) = 5.11, *p* = .009, η_p_^2^ = .13). Post-hoc Tukey’s comparisons (all *ps <* .05) revealed that children shared more with E1 in the *Baseline* condition (*M =* 72.00) compared to both the *Failed Commitments—Negative Outcomes* (*M =* 50.64) and *Failed Commitments—Positive Outcomes* conditions (*M =* 49.40), (See Fig 3).

### Epistemic decisions (ask and endorse trials)

#### Ask trials

**Fig 4:**
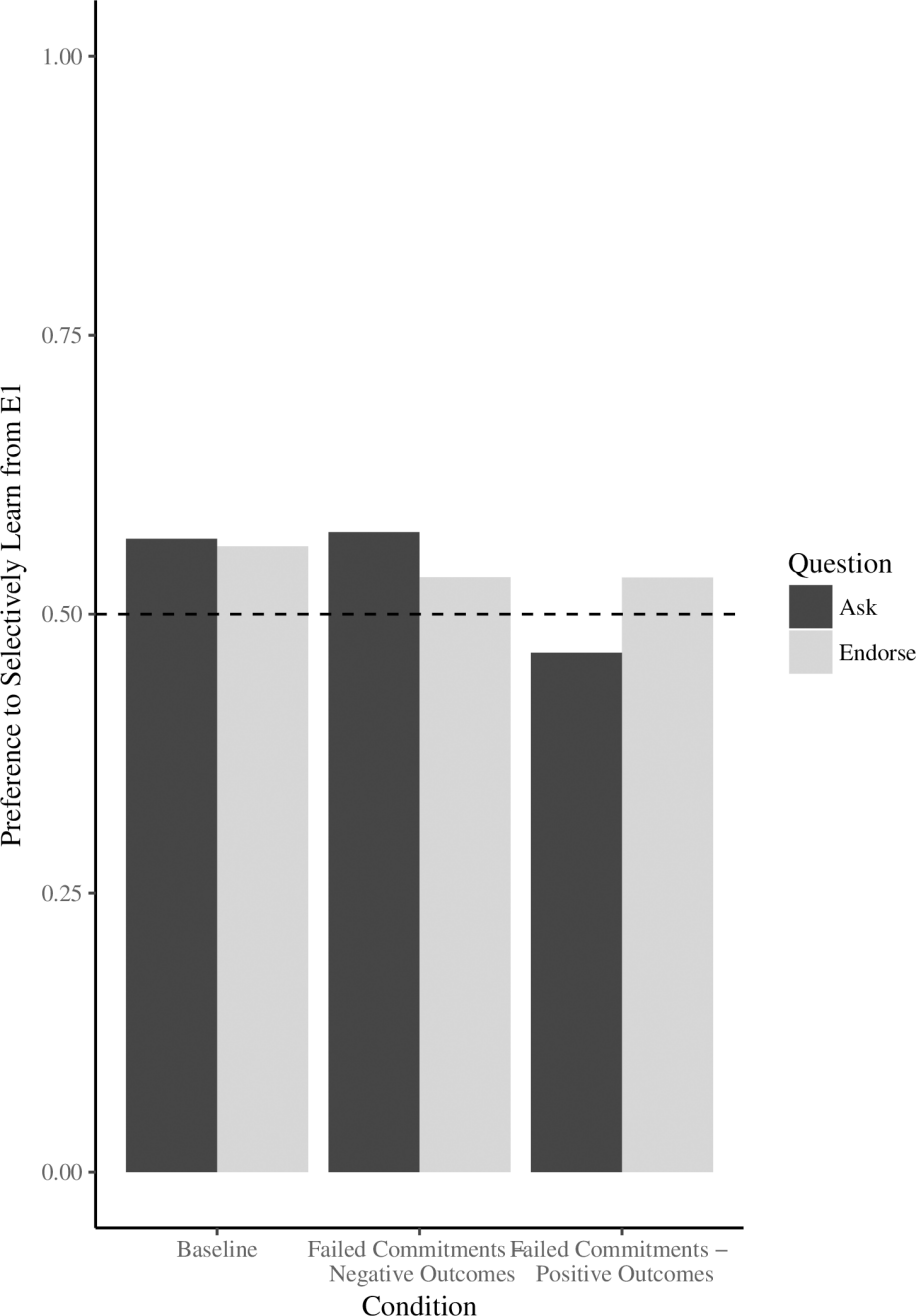
Mean proportion of times participants asked or endorsed E1 (out of 6 trials each) by condition. Dashed line represents chance. Error bars represent standard errors of the mean.

An ask score was calculated by summing the amount of trials out of six that children chose E1 as the person they would like to ask to find out where an object was hidden. This score was converted to a percentage for ease of interpretation. An omnibus ANOVA was run with age (3 vs. 4) and condition (*Baseline* vs. *Failed Commitments—Negative Outcomes* vs. *Failed Commitments—Positive Outcomes*) as between-subjects factors, and ask score as the dependent measure. We found a non-significant, but trending effect of age (*F*(1,68) = 3.18, *p* = 0.07, η_p_^2^ = .04), with 4-year-olds somewhat more likely to ask E1 for information (*M* = .58) compared to 3-year-olds (*M =* .48). We found no effect of condition (*F*(2,68) = 1.75, *p* = 0.18) and no interaction between age and condition (*F*(2,68) = 1.21, *p* = 0.31) (See Fig 4).

#### Endorse trials

An endorse score was calculated by summing the amount of trials out of six that children chose to endorse the location offered by E1. An omnibus ANOVA was run with age (3 vs. 4) and condition (*Baseline* vs. *Failed Commitments—Negative Outcomes* vs. *Failed Commitments—Positive Outcomes*) as between-subjects factors, and endorse score as the dependent measure. There were no significant differences between conditions (*F*(2,69) = 0.12, *p* = 0.88) or between age groups (*F*(1,69) = 1.43, *p* = 0.23) and no interaction between age and condition (*F*(2,69) = 0.61, *p* = 0.54) (See Fig 4).

## Discussion

Much interest in testimonial learning has focused on children’s selective *trust* in testimony; however, the precise nature of this trust remains unknown. In the current study, we presented children with one kind of interpersonally bad agent–one who invoked an interpersonal violation of trust by failing to deliver a desired item or one who failed to withhold a desired item. These failures both reflect failed commitments that were stated directly to participants. We found that this manipulation reduced 3- and 4-year-olds’ decisions to wait for and share with E1, but did not similarly reduce their decisions to seek or endorse information offered by her. To account for this dissociation, we suggest distinguishing between cases of ‘epistemic or evidential trust’ from ‘interpersonal trust.’ In cases of *epistemic trust*, decisions to accept what someone claims are responsive to different types of inductive evidence that bear on the claim (e.g. such as the listener’s prior knowledge and/or the speaker’s certainty, prior reliability, their expertise, moral behavior, etc.). In cases of *interpersonal trust*, when we trust someone who made a commitment or promise to us, we do so not because we have collected the evidence (after all, whether they keep their commitment is up to them, not us) [24, 27]. Rather, when a speaker makes a commitment to act in a specific way to a listener, she offers an answer to the question of what she will do. If the listener accepts her answer in the spirit in which it is given, the listener has decided to *trust* the speaker interpersonally, taking her at her word.

Our findings argue for identifying epistemic reasons to trust in testimony that are based on weighing the evidence one has to come to a conclusion (evidence about the world, the agent, etc.) as distinct from interpersonal decisions to trust, which can be based on equally compelling reasons to trust that person (e.g., speech acts like promises, relations between speaker and addressee). This idea is supported by other studies which have manipulated agent characteristics across multiple domains. For example, when introduced to characters who differed in niceness and expertise, children based epistemic inferences on evidence of both expertise and niceness but based social inferences only on niceness [35]. Similarly, when introduced to in-group members who refused to share, children’s practical decisions to share were reduced but their epistemic decisions to learn from that agent were not [33]. These findings suggest that children appreciate that an agent can be untrustworthy for interpersonal reasons (e.g., relationship-building) but still have knowledge to share. We build upon this work in the present study by examining how a speaker’s interpersonal commitments influence a range of decisions, both practical and epistemic. Given recent evidence that a delay of gratification task measures children’s *trust* [31], our manipulation suggests that in our study, and in other similar experiments (e.g. [33]), a distinct type of trust was reduced, one that may not be implicated in all testimonial learning decisions. *Trusting* someone does not consist solely in predicting their reliability in light of the evidence we have collected. In fact, reasons to trust a person can be quite distinct from evidential reasons to favor someone’s testimony [27, 45]. Thus, it is important to present children with a range of decisions involving multi-dimensional agents in order to better understand when and how reasons to trust a person might dissociate from reasons to believe a claim.

Consistent with these ideas is the possibility that certain speech acts (promising, requesting, resolving) instigate children’s decisions to trust, while other speech acts (arguments, explanations, demonstrations) do not. As discussed above, when we accept an agent’s spoken commitments *on trust* as the answer to the question of what she will do, we accept her answer as our conclusion [27]. Explanations or arguments, in contrast, obviate the need for trust by placing children in a position to evaluate a set of considerations and draw conclusions based on them. In the current study, if children treated an agent’s commitments as direct bids for trust, then children’s distrust on delay tasks and sharing tasks can be seen as direct, natural responses to an experimenter who made a commitment, but who failed to uphold that commitment, thereby violating the trust invoked by it. Moreover, the current study highlights that failed commitments, even when they produce positive outcomes, can reduce children’s trust in that speaker. This suggests that children register not only when others let them down or disappoint them, but also when others commit to a negative action, punishment, or “threat” that they did not execute. The inconsistencies between one’s commitments and one’s actions may produce breaches of interpersonal trust.

Our learning trials, which presented speakers’ divergent claims about object locations, were not responsive to the manipulation. In contrast to delay decisions and resource allocations, children’s performance did not differ from chance in any of the three conditions. This may reflect the absence of interpersonal trust in the testimonial learning decisions that we presented, trials for which other kinds of evidence (e.g., perceptual access) may have been privileged [46, 6] However, there are other explanations for this null finding. One possibility is that because our learning trials did not present speakers who made promises or stated commitments, and did not present interpersonal bids for trust per se, children did not treat their prior experiences with the experimenter as relevant to the decisions posed by the learning trials. Along these lines, perhaps children simply treated the experimenter’s variable commitment-keeping behavior as *evidence* for or against her reliability. If true, however, it goes unexplained why this evidence about reliability was not registered in their evaluations of her testimony. In other words, a strictly evidential picture of children’s trust leaves it unclear why children were selective in their practical decisions (to wait, and to share) but not their epistemic decisions (to ask, and to endorse). After all, the experimenter showcased both inaccurate claims (negative outcomes) as well as lack of goodwill (negative intentions), both of which are forms of evidence known to attenuate children’s learning decisions [11, 12, 32]. If children reduced an agent’s failed commitments to evidence, it will need to be explained (a) why the agent’s unreliability was registered in certain practical decisions and not epistemic ones, and (b) why the interpersonal reasons to trust invoked by commitments played no role.

Another possibility is that children extend a form of interpersonal trust when considering a broad range of communication, including the claims made by speakers on our learning trials, and our manipulation was not sufficient to disrupt the default levels of trust that children regularly extend [47]. Indeed, our experimental manipulation ensured that children did not receive information about the differential knowledge or access of our two agents, suggesting that in the absence of conflicting epistemic information, children may have found both agents worth trusting for new information. Consistent with this is the possibility that the mechanisms underlying children’s decisions to trust in testimony may not be exclusively responsive to epistemic or evidential considerations, but interpersonal reasons as well. By taking into account when and how interpersonal bases for trust affect learning decisions, we can begin to expand current evidential models of selective trust and better discriminate the kinds of reasons children have to trust others. While children’s lack of selective learning in the present study could be due to the absence or presence of interpersonal trust, it nonetheless contrasts with the selectivity produced by the practical decisions children made. Future work will be necessary to understand how interpersonal trust functions in epistemic and practical decisions.

Current research in children’s testimonial learning seeks to take seriously the varieties of trust that feature in children’s cognitive development [48]. Recent work has begun to shift attention towards the various social goals [49] or inferences [18] that might feature in children’s selective learning from others (see also [50, 51]). Our findings demonstrate a dissociation between children’s practical and epistemic decisions and call for closer examination of the forms of trust that feature in such decisions. In addition, it will be important for future work to distinguish between epistemic and interpersonal bases for trust in samples from more diverse populations. For example, children growing up in less reliable environments may be less trusting in general, or more uniquely vigilant of certain violations of trust. We also think it important for future work to consider the possibility that when children accept information from deceptive informants (e.g. [47, 52]), it may not reflect irrational decisions that neglect the evidence, but rather children’s robust decisions to trust another person for interpersonal reasons, despite evidence against them.

## Supporting information

S1 File Experimental script: Order 1A. (PDF)

S2 File Experimental script: Art project and delay of gratification. (DOCX)

S1 Dataset (XLSX)
