# Supplementary material for: Varieties of trust in preschoolers’ learning and practical decisions
Source: PLoS One. 2018 Aug 20;13(8):e0202506. doi: 10.1371/journal.pone.0202506 (PMC6101396; doi:10.1371/journal.pone.0202506)
Supplement: S1 File — (PDF) [file pone.0202506.s001.pdf]

Subject #_________ DOB: ____________ Today's Date: __________ Age: ________ Gender: ________

Order 1A: Familiar speaks 1st, Familiar on Left

MOOD RATING 1: **Bring out scale 1.** Look at these smiley faces. This one is the most happy, this one is the least happy, and these are in between. Circle the smiley face that matches how happy you are right now!

____

EXPLICIT LIKING 1: **Bring out scale 2.** "Remember this? Now I'm going to ask you some questions using this scale. Remember, on this scale, you can point to here if you really like something, here for like, kind of like, kind of don't like, don't like, or really don't like. Okay?" Start video and pause on still image of FAMILIAR and say, "Remember her?"

FAMILIAR: "How much do you like her?" ______

NEUTRAL: "How much do you like her?" ______

LEARNING/RES ALLOC: "See these people? These people are going to tell us things. I have a bunch of boxes where things are hidden and I want you to listen very carefully to what these two people say and then I'm going to ask you where you think the hidden things are, ok?"

TRIAL 1

ASK 1: "The first think that's hiding is this key. I wonder where it is!
Who would you like to ask to find out?" FAMILIAR NEUTRAL
"Let's see what they say."

FAMILIAR: "I think it's in the blue box."
NEUTRAL: "I think it's in the red box." **E: Repeat what each person said.**

ENDORSE 1: "Where do you think the key is?" BLUE RED

TRIAL 2

ASK 2: "The next thing that's hiding are these glasses. I wonder where it is!
Who would you like to ask to find out?" FAMILIAR NEUTRAL
"Let's see what they say."

NEUTRAL: "I think it's in the green box"
FAMILIAR: "I think it's in the yellow box" **E: Repeat what each person said.**

ENDORSE 2: "Where do you think the glasses are?" YELLOW GREEN

RES ALLOC 1: "Here are five coins for you to share. How many coins should this person [FAMILIAR] get and how many coins should this person [NEUTRAL] get?"

FAMILIAR __________ NEUTRAL __________

TRIAL 3

ASK 3: "The next thing that's hiding is this pencil. I wonder where it is!
Who would you like to ask to find out?" FAMILIAR NEUTRAL
"Let's see what they say."

FAMILIAR: "I think it's in the brown box"
NEUTRAL: "I think it's in the white box" **E: Repeat what each person said.**

ENDORSE 3: "Where do you think the pencil is?" BROWN WHITE

Subject #_________ DOB: ____________ Today's Date: __________ Age: ________ Gender: ________

TRIAL 4

ASK 4: "The next thing that's hiding is this spoon. I wonder where it is!
Who would you like to ask to find out?" FAMILIAR NEUTRAL
"Let's see what they say."

NEUTRAL: "I think it's in the orange box"
FAMILIAR: "I think it's in the black box" **E: Repeat what each person said.**

ENDORSE 4: "Where do you think the spoon is?" BLACK ORANGE

RES ALLOC 2: "Here are five coins for you to share. How many coins should this person [NEUTRAL] get and how many coins should this person [FAMILIAR] get?"
FAMILIAR __________ NEUTRAL __________

TRIAL 5

ASK 5: "The next thing that's hiding is this ball. I wonder where it is!
Who would you like to ask to find out?" FAMILIAR NEUTRAL
"Let's see what they say."

FAMILIAR: "I think it's in the yellow box"
NEUTRAL: "I think it's in the blue box" **E: Repeat what each person said.**

ENDORSE 5: "Where do you think the ball is?" YELLOW BLUE

TRIAL 6

ASK 6: "The next thing that's hiding is this watch. I wonder where it is!
Who would you like to ask to find out?" FAMILIAR NEUTRAL
"Let's see what they say."

NEUTRAL: "I think it's in the red box"
FAMILIAR: "I think it's in the green box" **E: Repeat what each person said.**

ENDORSE 6: "Where do you think the watch is?" GREEN RED

RES ALLOC 3: "Here are five coins for you to share. How many coins should this person [FAMILIAR] get and how many coins should this person [NEUTRAL] get?"
FAMILIAR __________ NEUTRAL __________

EXPLICIT LIKING 2: **Bring out scale 2.** "Remember this? Now I'm going to ask you some questions using this scale. Remember, On this scale, you can point to here if you really like something, here for like, kind of like, kind of don't like, don't like, or really don't like. Okay?"
NEUTRAL: "How much do you like her?" ______
FAMILIAR: "How much do you like her?" ______

MOOD RATING 2: **Bring out scale 1.** Look at these smiley faces. This one is the most happy, this one is the least happy, and these are in between. Circle the smiley face that matches how happy you are right now!

____
