# Supplementary material for: Varieties of trust in preschoolers’ learning and practical decisions
Source: PLoS One. 2018 Aug 20;13(8):e0202506. doi: 10.1371/journal.pone.0202506 (PMC6101396; doi:10.1371/journal.pone.0202506)
Supplement: S2 File — (DOCX) [file pone.0202506.s002.docx]

Subject #_________ DOB: ____________ Today’s Date: __________ Age: ________ Gender: ________

**Condition 1: Positive Intention, Positive Outcome**

**DELAY OF GRATIFICATION**

*Have cardstock and used crayons and stickers on table in testing room.*

“So, today we have a very exciting art project planned for you! In the other room, we have everything we’ll need for you to make your own picture like this one! And you’ll be able to take it home with you! Does that sound like something you’d like to do?”

*Walk to testing room*

**CHOICE 1:**

“To decorate your picture, you have a choice of what art supplies to use. You could use these [crayons] right now. Or—if you can wait for new supplies from the other room—you can use a better set of art supplies instead. The best set has markers, pens, colored pencils—a lot of cool stuff. How does that sound?” [Response.]

“Okay, I really want you to have the best art supplies. I’m going to go and you should stay right here in that chair until I come back.”

*Allow 2.5 minutes to elapse and return*

“Here is the best set of art supplies!”

*Allow/encourage child to draw for 2 minutes*

**CHOICE 2:** *Produce a round 1/4-in. reward-style sticker from pocket sealed inside of a plastic envelope*

**“**Would you like to add a sticker to your picture? [Response.] For stickers, you have a choice. You can use this [sticker] right now. Or—if you can wait for new stickers from the other room—you can have a bunch of stickers to use instead. How does that sound?” [Response.]

“Okay, I really want you to have the best stickers. I’m going to go and you should stay right here in that chair until I come back.”

*Allow 2.5 minutes to elapse and return*

“Here are the best stickers!”

*Allow/encourage child to draw for 2 minutes*

**TEST PHASE:**

“You finished just in time, because now it’s snack time! You have a choice for your snack. You can eat this one marshmallow right now. Or—if you can wait for me to go get more marshmallows from the other room—you can have two marshmallows to eat instead. How does that sound?” [Response.]

“Okay, I’m going to leave and you should stay right here in that chair until I come back. Can you do that? [Response.] I’ll leave this [marshmallow] here, and if you haven’t eaten it when I come back, you can have two marshmallows instead!”

*Place the marshmallow directly in front of the child, 4 in. from the table’s edge. Quickly collect the art materials and drawing and leave the room until either: A) child consumes the marshmallow or B) until 10 min has elapsed.*

Subject #_________ DOB: ____________ Today’s Date: __________ Age: ________ Gender: ________

**Condition 2: Positive Intention, Negative Outcome**

**DELAY OF GRATIFICATION**

*Have cardstock and used crayons and stickers on table in testing room.*

“So, today we have a very exciting art project planned for you! In the other room, we have everything we’ll need for you to make your own picture like this one! And you’ll be able to take it home with you! Does that sound like something you’d like to do?”

*Walk to testing room*

**CHOICE 1:**

“To decorate your picture, you have a choice of what art supplies to use. You could use these [crayons] right now. Or—if you can wait for new supplies from the other room—you can use a better set of art supplies instead. The best set has markers, pens, colored pencils—a lot of cool stuff. How does that sound?” [Response.]

“Okay, I really want you to have the best art supplies. I’m going to go and you should stay right here in that chair until I come back.”

*Allow 2.5 minutes to elapse and return*

“I’m sorry but we don’t have any other art supplies after all. Why don’t you use these instead?”

*Allow/encourage child to draw for 2 minutes*

**CHOICE 2:** *Produce a round 1/4-in. reward-style sticker from pocket sealed inside of a plastic envelope*

**“**Would you like to add a sticker to your picture? [Response.] For stickers, you have a choice. You can use this [sticker] right now. Or—if you can wait for new stickers from the other room—you can have a bunch of stickers to use instead. How does that sound?” [Response.]

“Okay, I really want you to have the best stickers. I’m going to go and you should stay right here in that chair until I come back.”

*Allow 2.5 minutes to elapse and return*

“I’m sorry but we don’t have any other stickers after all. Why don’t you use this instead?”

*Allow/encourage child to draw for 2 minutes*

**TEST PHASE:**

“You finished just in time, because now it’s snack time! You have a choice for your snack. You can eat this one marshmallow right now. Or—if you can wait for me to go get more marshmallows from the other room—you can have two marshmallows to eat instead. How does that sound?” [Response.]

“Okay, I’m going to leave and you should stay right here in that chair until I come back. Can you do that? [Response.] I’ll leave this [marshmallow] here, and if you haven’t eaten it when I come back, you can have two marshmallows instead!”

*Place the marshmallow directly in front of the child, 4 in. from the table’s edge. Quickly collect the art materials and drawing and leave the room until either: A) child consumes the marshmallow or B) until 10 min has elapsed.*

Subject #_________ DOB: ____________ Today’s Date: __________ Age: ________ Gender: ________

**Condition 3: Negative Intention, Positive Outcome**

**DELAY OF GRATIFICATION**

*Have cardstock and used crayons and stickers on table in testing room.*

“So, today we have a very exciting art project planned for you! In the other room, we have everything we’ll need for you to make your own picture like this one! And you’ll be able to take it home with you! Does that sound like something you’d like to do?”

*Walk to testing room*

**CHOICE 1:**

“To decorate your picture, you have a choice of what art supplies to use. You could use these [crayons] right now. Or—if you can wait for new supplies from the other room—you can use a better set of art supplies instead. The best set has markers, pens, colored pencils—a lot of cool stuff. How does that sound?” [Response.]

“Actually, I really don’t want you to have the best art supplies because I want the best for myself. I’m going to go and you should stay right here in that chair until I come back.”

*Allow 2.5 minutes to elapse and return*

“Actually, here is another set of supplies that you could use. I saved the VERY best set for myself, but you can use these.”

*Allow/encourage child to draw for 2 minutes*

**CHOICE 2:** *Produce a round 1/4-in. reward-style sticker from pocket sealed inside of a plastic envelope*

**“**Would you like to add a sticker to your picture? [Response.] For stickers, you have a choice. You can use this [sticker] right now. Or—if you can wait for new stickers from the other room—you can have a bunch of stickers to use instead. How does that sound?” [Response.]

“Actually, I really don’t want you to have the best stickers because I want the best for myself. I’m going to go and you should stay right here in that chair until I come back.”

*Allow 2.5 minutes to elapse and return*

“Actually, here is another set of stickers that you could use. I saved the VERY best stickers for myself, but you can use these.”

*Allow/encourage child to draw for 2 minutes*

**TEST PHASE:**

“You finished just in time, because now it’s snack time! You have a choice for your snack. You can eat this one marshmallow right now. Or—if you can wait for me to go get more marshmallows from the other room—you can have two marshmallows to eat instead. How does that sound?” [Response.]

“Okay, I’m going to leave and you should stay right here in that chair until I come back. Can you do that? [Response.] I’ll leave this [marshmallow] here, and if you haven’t eaten it when I come back, you can have two marshmallows instead!”

*Place the marshmallow directly in front of the child, 4 in. from the table’s edge. Quickly collect the art materials and drawing and leave the room until either: A) child consumes the marshmallow or B) until 10 min has elapsed.*
